# Decoding Adolescents’ and Parents’ Perspectives of Overeating: A Qualitative Study

**DOI:** 10.3390/bs16030328

**Published:** 2026-02-27

**Authors:** Kirrilly M. Pursey, Hiba Jebeile, Deborah Mitchison, Janelle A. Skinner, Natalie B. Lister, Megan Whatnall, Mark Leary, Tracy L. Burrows

**Affiliations:** 1 College of Health, Medicine and Wellbeing, University of Newcastle, Newcastle, NSW 2308, Australia; 2 Food and Nutrition Program, Hunter Medical Research Institute, University of Newcastle, New Lambton Heights, NSW 2305, Australia; 3 Children’s Hospital Westmead Clinical School, Faculty of Medicine and Health, University of Sydney, Westmead, NSW 2145, Australia; 4 Discipline of Clinical Psychology, Graduate School of Health, University of Technology, Sydney, NSW 2007, Australia; 5 School of Health Sciences, University of Newcastle, Callaghan, NSW 2308, Australia

**Keywords:** adolescence, overeating, binge eating, help-seeking, stigma, eating behaviours, Integrated Knowledge Translation

## Abstract

Objective: Adolescence is a high-risk period for problematic eating behaviours, including overeating. However, few studies have explored adolescent perceptions of these eating behaviours and whether there is a shared understanding between adolescents and parents. This study aimed to investigate perceptions of eating behaviours, focusing on overeating, in Australian adolescents and parents. Method: Adolescents aged 13–19 years, and parents of adolescents, participated in two interviews for exploration and thematic deepening of participant perceptions, underpinned by Integrated Knowledge Translation Framework principles. Interviews explored perceptions of overeating and other eating behaviours, including help-seeking and stigma. Data were analysed thematically. Results: Twelve adolescents (59% female) and seven parents (100% female) participated in the interviews, with three major themes emerging. In theme 1, “perceptions of overeating”, interpretations of overeating varied; however, both adolescents and parents associated problematic overeating with increased frequency and impacts on functioning. Discrepancies between adolescent and parent perceptions of overeating terms such as binge eating were present. In theme 2, “beliefs about overeating”, adolescents felt that broaching the topic of overeating and help-seeking for overeating to be more challenging than restrictive eating disorders due to stigma. In theme 3, “perceptions of other eating behaviours”, there were differences between how adolescents perceived healthy eating and dieting compared to parents. Discussion: Differences in adolescent and parent understanding of eating behaviour terminology highlights a need for a shared language to support appropriate detection of problematic eating behaviours. There is a need for prevention and early intervention approaches that promote awareness and accessible support pathways for overeating to prevent progression to an eating disorder.

## 1. Introduction

Adolescence is an important and transformative period of growth and development, characterised by a range of cognitive, physical, and psychosocial changes. Adolescence is a high-risk period for the development of problematic eating behaviours, as many psychological and physical changes occur during this period (Pringle et al., 2016). Many mental health issues, including eating disorders (ED), also first present during adolescence (Smink et al., 2012). Importantly, eating behaviours developed during childhood and adolescence typically track into adulthood (Appannah et al., 2021), highlighting this as an important life stage to establish healthy behaviours. As young adults have the most negative health trajectory of any population group, early intervention in problematic eating behaviours during adolescence is paramount to prevent future morbidity and mortality and improve overall population health (Truesdale et al., 2006; Zheng et al., 2016).

Overeating is an eating behaviour that overlaps with health conditions such as higher weight, mental illness, and disordered eating behaviours, particularly binge eating and bulimia nervosa. Adolescents experiencing overeating may therefore present for treatment at a range of clinical or health services. However, given the complexity of overeating, its overlap with other conditions, and the siloing of many mental and physical health services, individuals may not receive effective treatment. One of the barriers to developing effective assessment, treatments, and service models for adolescents who experience overeating is the lack of specific evidence around adolescents’ perceived treatment needs and understanding of terminology, which may influence how effectively screening and assessment tools capture these behaviours. This is important as disordered eating behaviours are common amongst Australian adolescents (Mitchison et al., 2020), and eating disorders where overeating is a core feature, such as binge eating disorder, are growing more rapidly than any other ED (Hoek, 2016), highlighting the need for appropriate recognition and care for these conditions.

A range of terms can be used by health professionals to assess, describe, and manage different eating behaviours, including overeating. This diversity in terminology and its interpretation may differ by individual, setting (i.e., weight management or mental health), and across common survey tools to assess eating behaviours, such as the Eating Disorders Examination Questionnaire (EDEQ) (Mond et al., 2004), and Yale Food Addiction Scale (YFAS) (Gearhardt et al., 2016). Often, eating behaviour terms change usage and meaning over time and are used interchangeably in research, by clinicians, and the community more broadly. For example, overeating: eating a large amount of food on an occasion, compared to binge eating: eating a large amount of food over a short period of time with or without loss of control, reflecting potentially divergent views of these terms. This has implications for the reliability of informant responses to assessment instruments in research and clinical practice. In addition, understanding perceptions of other eating behaviours (e.g., healthy eating, restrictive eating) may assist in comparing views of overeating to other eating behaviours.

Given the considerable cognitive development and increasing independence during adolescence, and generational differences in the use of language, there is also the potential for differences in perceptions of eating behaviours between adolescents and their parents or caregivers. Previous studies have reported discrepancies between adolescent and parent perceptions of health communication (Wecht et al., 2024), which can negatively impact adolescent psychological health (Kapetanovic & Boson, 2022). It is unclear if adolescents and their families have a shared understanding of eating behaviours, including overeating, and associated terms, or if some terms are better understood than others. In clinical care, parents may or may not be involved in adolescent care in certain situations, depending on the maturity of the child and the care situation. Further, clinicians often rely on parents as an additional informant for collateral perspectives in clinical assessment, and the main treatment models for EDs and weight management often involve family-based sessions. A better understanding of eating behaviours, including overeating, across adolescents and their families is therefore needed to strengthen existing service delivery models.

Currently, few clinical services are available that specifically target overeating behaviours in adolescents. Most adolescent ED models focus on conditions that are associated with extreme weight control behaviour, with overeating often a secondary focus, if at all. As older adolescents are nearing age cut-offs for paediatric services, adult models of care are often applied, although their suitability for adolescents is unclear. In addition, overeating may be uniquely silenced or more commonly framed as a lack of control and internalised by the individual compared to other problematic eating behaviours, particularly restrictive disorders, which may influence help-seeking. In addition, binge eating disorder is more common in those of a higher weight and remains highly stigmatised in the general community (Côté et al., 2025). However, few studies have investigated the perceived stigma of overeating compared to restrictive eating behaviours. It is important to better understand perceptions of eating behaviours and associated terminology to inform how we discuss, assess, and identify eating behaviours as well as the need for intervention, and to inform health promotion, early intervention, and treatment strategies that connect to their lived experience. This is particularly important given the rise in prevalence of disordered eating in those at higher weights (da Luz et al., 2017), and findings that ED experienced during adolescence are more prevalent in those who have age and sex adjusted body mass index (BMI) percentiles > 85 (Mitchison et al., 2020).

This novel project explores the perceptions of eating behaviours, with a specific focus on overeating, in adolescents and caregivers, including associated terminology and help-seeking perceptions. The findings of this study will assist in identifying potential discrepancies in nomenclature and service gaps in addressing overeating in this group, which is currently lacking, to improve engagement and equitable access to treatment and early intervention opportunities for adolescents experiencing overeating behaviours. This study, therefore, aimed to investigate perceptions of eating behaviours, with a particular focus on overeating behaviours, in Australian adolescents aged 13–19 years and parents of adolescents.

## 2. Materials and Methods

### 2.1. Study Design

The qualitative research approach of interpretive description (Thorne et al., 2004), underpinned by iKT principles, was used to gain understanding of the phenomenon of overeating through the perspectives of adolescents aged 13–19 years and parents or caregivers of adolescents (i.e., ‘knowledge users’). The iKT approach involves targeted engagement and collaboration with those who will use the knowledge derived from the research (Graham et al., 2019), for example, consumers and the general public. The iKT framework was selected for this study to collaborate with adolescents and parents to capture subjective perceptions of overeating and other eating behaviours, as well as treatment and help-seeking needs, to generate an interpretive description to inform clinical understanding and future care. Two interviews were conducted with adolescents and parents in line with the iKT framework. We also considered the cyclic obesity/weight-based stigma (COBWEBS) conceptual lens for the interpretive stance relating to stigma-related data in the paper, as well as community eating disorder mental health literacy (Mond, 2014). This study was approved by the University of Newcastle Human Research Ethics Committee (Approval Number H-2021-0279). The reporting of this study adheres to the Consolidated Criteria for Reporting Qualitative Studies (COREQ) Checklist. (Tong et al., 2007).

### 2.2. Participants and Recruitment

The knowledge users recruited to the interviews included two groups to gain a wide range of perceptions: (i) adolescents with or without lived experience of overeating, and (ii) parents of adolescents. Participants were eligible to participate if they were adolescents aged between 13 and 19 years, or parents or caregivers of an adolescent aged 13–19 years, who had access to the internet, and were proficient in the English language. Adolescents and parents or caregivers did not have to be matched dyads, i.e., the parents or caregivers of participating adolescents. Potential participants were a convenience sample identified via professional networks of clinicians working across weight management, eating disorders, and mental health settings. Individuals who had participated in previous research studies conducted by the research team who elected to be recontacted for future nutrition-related research were also invited.

Potential participants were emailed and provided with a link to the online participant information sheet and demographic screening survey in REDCap, a secure electronic data capture tool (Harris et al., 2009). The demographic information collected from participants included gender and age category (adolescents: 13–15, 16–17, 18–19 years; parents/caregivers: 30–39, 40–49, 50–59 years) and a multiple choice question regarding their experience and familiarity of problematic eating behaviours, ED or body image concerns (response options included previous participation in a study, lived experience, a friend or relative affected by this, reading about it, or do not wish to disclose). Upon completion, a member of the research team then contacted eligible participants to schedule a time for their initial interview. Eligible adults provided written consent, and adolescents provided written agreement with parental consent to participate. Adolescents attended interviews independent of parents. The first interviews were held between January and May 2022, and the second interviews between October and December 2022. Participants were offered a gift card (20AUD) for their participation in each interview of the study.

### 2.3. Data Collection

Data collection involved two semi-structured interviews (Stage 1 and Stage 2). The interview schedules were developed by the research team with clinical and research experience in dietetics, mental health, and eating disorders. The Stage 1 interviews were approximately 60 min in length. The Stage 1 interview schedule included questions around familiarity of terms associated with overeating commonly used by health professionals and screening or assessment tools (e.g., overeating, binge eating, night eating, food addiction), as well as help-seeking attitudes towards overeating behaviours; sources of help or treatment for overeating to optimally engage adolescents; perceived barriers and facilitators to treatment or seeking help for treatment; and perceived stigma around overeating behaviours (Supplementary Table S1). To contrast perceptions of overeating, questions relating to other eating behaviours, including healthy eating and restrictive eating, were asked. For adolescents, questions were asked of themselves and their peers, and for parents/caregivers, this was asked in reflection of their child or their peers. Interview questions were piloted by researchers and clinicians, with minor refinements made to question wording.

Consistent with the iKT framework that promotes collaboration, a second interview was completed (approx. 2–5 months after Stage 1) to increase mutual understanding between researchers and participants. Participants in Stage 2 were sent a summary of the preliminary themes prior to the second interview for clarification, thematic deepening, and further detail in the Stage 2 interview. It also included questions that the research team deemed worthy of greater exploration following preliminary consensus discussions of the Stage 1 interviews (for example, stigma associated with overeating). The Stage 2 interviews were approximately 30 min in length.

The interviews were conducted online via Zoom videotelephony software, Version 6.6.6 (Zoom Video Communications Inc, San Jose, CA, USA). This allowed participants to participate in a location of their choice, most often their home environment. Interviews were facilitated by a female interviewer who was an experienced clinical or PhD-qualified dietitian working with overeating and adolescent populations (TB and SH), as well as training in qualitative methods enquiry. The interviewers had no prior relationship with the participants, and at the start of the first interview, they provided their background as dietitians to the participants. Individual interviews were audio recorded and transcribed verbatim; the interviewer also kept field notes.

### 2.4. Data Analysis

An inductive thematic analysis (Thomas, 2006) was conducted and worked toward the two main tasks of data analysis in interpretive description (Thorne et al., 2004), specifically ‘identification of themes within coding categories’ and ‘identification of themes across coding categories’ (Knafl & Webster, 1988) to characterize the phenomenon of overeating and contextualize how these experiences play out in day-to-day situations. Since the aim of the study was explorative in nature, elucidating the variations in perceptions of the knowledge users, a thematic analysis was deemed most appropriate. During this study, the process of constant comparative analysis (Glaser & Strauss, 1967; Thorne et al., 2004) was used, meaning that, after the Stage 1 interviews, data analysis was completed prior to commencing Stage 2, allowing for the use of the newly acquired data in the Stage 2 interviews.

In the first phase of the analysis, transcripts of Stage 1 interviews were reviewed by two dietetic researchers (JS and EB) to gain a general understanding of the meaning and then coded independently. After the initial coding, researchers revised the codes until consensus on definitive codes was reached to develop themes. The data were tabulated and grouped into themes by two reviewers (JS and EB), with discussion occurring between reviewers to thoroughly examine interpretations by comparing themes and how they interrelate. The themes were also compared to the interviewer’s field notes to check the first impressions and what became apparent through the transcribed words. To obtain feedback on interpretations during data analysis, the data were discussed with the broader iKT research group, after which the central recurring themes were extracted from the data, defined, and named. Emerging themes were documented, followed by research team meetings to reach agreement on emerging themes and topics. Follow-up questions were then developed for the Stage 2 interviews by the research group to further explore the themes that emerged in more detail. Stage 2 interviews were coded by a single dietetic researcher (KP). Credibility checking of coding of the second interviews was achieved via multiple team members reviewing interviews and discussion of coding and themes among group members (KP and TB). The interviewers and coders kept reflexive notes throughout the interview and coding process, and discussed with other members of the team to keep a self-critical account of professional and personal experiences throughout the study process. The research group consisted of five individuals who were academics and clinicians from both a dietetic and psychology background, all of whom had research and/or clinical experience with disordered eating. The arising themes were used in the descriptive model, as it is presented in the results section of this paper.

Trustworthiness was enhanced in the following ways: (i) careful triangulation between the interviews, the interview transcriptions and field notes were used to cross verify the findings; (ii) a rich description of the participant’s understandings and perceptions are included in the results section with representative quotations from participants (A1–A12, adolescents; and P1–P7, parents or caregivers) to illustrate conceptual interpretation of the data; (iii) consensus was reached through research team discussions at each step of the analysis process; and (iv) the transcripts from each of the interviews were offered to be returned to participants for comment and/or correction.

## 3. Results

A total of twelve adolescents and seven parents completed the Stage 1 screening survey and interviews. Of the adolescents, most were female (*n* = 7), and in the 13–15 year age category (*n* = 7), followed by 16–17 years (*n* = 2) and 18–20 years (*n* = 3). Adolescents’ experience with problematic eating behaviours included ‘other’ with no further details (*n* = 8), relative/close contact affected by this (*n* = 2), participated in a previous study (*n* = 1), or read about it (*n* = 1). No adolescents reported that they themselves had experienced problematic eating behaviours. Of the parents/caregivers, all were female; in the 40–49 year (*n* = 5), or 50–59 years (*n* = 2) age categories. Parents/caregivers’ responses about their experience and familiarity with problematic eating behaviours included reading about it (*n* = 3), having a friend or family member affected by this (*n* = 2), and ‘other’ with no further details provided (*n* = 2). Seven adolescents and five adults completed the Stage 2 interviews. The main reason for not participating in the second interview was a lack of availability.

**Figure 1:**
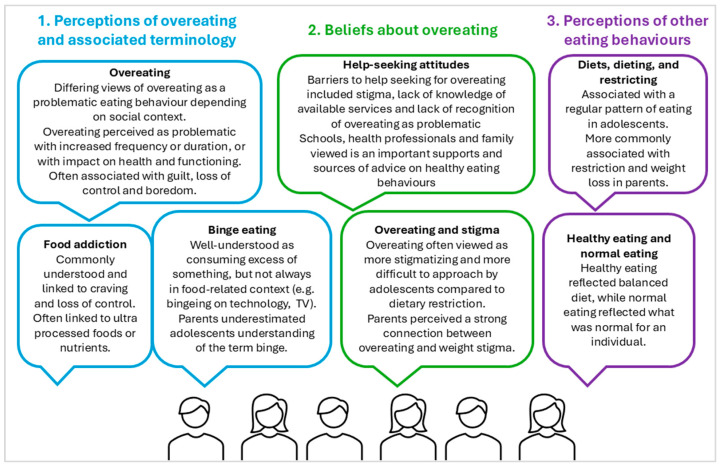
Summary of themes and sub-themes identified from the synthesis of stage one and two interviews. Main themes are in coloured text and sub-themes in colour-matched speech bubbles, including an explanation of the sub-theme in non-bolded text.

The research group discussed the Stage 1 themes and identified nine elements to be addressed and presented for clarification and thematic deepening of understanding during the Stage 2 interviews (Supplementary data). Qualitative analysis identified three major themes, with two subthemes each. Figure 1 presents the major themes and subthemes from the synthesis of the Stage 1 and 2 interviews.

### 3.1. Theme 1: Perceptions of Overeating and Associated Terminology

#### 3.1.1. Subtheme 1: Overeating

Across the adolescent respondents, there was a range of interpretations of overeating and associated terms. In adolescents, the term ‘overeating’ had conflicting views, with some linking to Diagnostic and Statistical Manual of Mental Disorders (DSM)-5 features of binge eating (e.g., guilt, eating past comfortably full, loss of control with eating, permissive and compensatory behaviours, boredom). For example, *“I would just keep going, and I don’t know when to stop, then afterwards [thinking] I went too far with that.”*—A1. However, other participants did not perceive overeating as problematic behaviour, with adolescents and parents viewing infrequent overeating sanctioned by social context, such as at parties, as part of normal eating. However, parents and adolescents perceived overeating as a problematic behaviour with increasing frequency or when symptoms become so severe they impact on health and functioning, for example, *“there’s a point where it might start off as [being] fun and then there’s a point where it’s way too much and it’s unhealthy”*—A2, and *“When it’s getting in the way of them living their life”*—A5.

In the Stage 2 of interviews, there were conflicting views across adolescent and parent interviewees of whether adolescents and parents would perceive overeating similarly, with one adolescent reporting their views may be different from their parents due to different exposures, including social media, *“Some of the same issues but I reckon it’s gotten worse for my generation because of social media”*—A2.

#### 3.1.2. Subtheme 2: Perceptions of Terms Associated with Overeating (Binge Eating, Food Addiction, and Night Eating)

The term ‘binge eating’ appeared to be associated with the DSM-5 definition of “a lot in a short amount of time” as well as linking to emotional responses such as feeling down, eating in secret, and eating when bored. Only one adolescent reported binge eating to be associated with loss of control. Adolescents also commonly reflected other uses of the word binge, for example, in relation to technology or drinking alcohol, terminology which appeared more commonly used and understood compared to eating. Few parents reported that adolescents would be familiar with the term ‘binge eating’, underestimating adolescents’ understanding of this. Paralleling the adolescent responses, in the Stage 2 parent interviews, the word ‘binge’ was perceived to be more commonly accepted by adolescents in relation to television programmes.

“Food addiction” was more commonly understood and consistently viewed by adolescents as a problematic eating behaviour compared to overeating in both the Stage 1 and Stage 2 interviews. “Food addiction” was also more commonly linked to specific types of food, particularly high-sugar foods. For example, *“It’s like an addiction to sugar… it’s like cigarettes”*—A6, *“Eating too much sugary foods and can’t really get off it”*—A3. Unlike binge eating, food addiction was more commonly associated with craving and loss of control, in line with features of other substance and behavioural addictions. Adolescents described food addiction as, *“Craving it all the time… Being hung up on it”*—A5, and *“Out of control, they can’t control themselves, if they see a certain food or see food they can’t control themselves or physically stop”*—A6. While parents reported that adolescents would be aware of the term ‘food addiction’, parents did not use the same terminology around the severity of food addiction, or aspects of craving and loss of control, with a simpler view of how they expected adolescents to understand this term, *“They understand what is meant by addiction, but would never put food with that”*—P1. In addition, compared to other types of overeating, the term ‘night eating’ was not widely recognized by adolescents or parents, associating this term with one-off eating at night.

### 3.2. Theme 2: Beliefs About Overeating

#### 3.2.1. Subtheme 1: Help-Seeking Attitudes

Barriers to seeking help for overeating for adolescents in Stage 1 are largely centred around stigma around weight and overeating, with these being perceived as sensitive topics. It was often reported by the adolescents as a difficult subject to raise if there was an issue as this may be perceived as embarrassing or *“Secrecy, restricting and bingeing is shameful because… [if you are] unhappy with yourself and you don’t want people to know what you are doing so getting help is hard and not accessible to know what steps to follow”*—A8. Inadequate understanding of whether overeating is problematic and limited knowledge of available services and access points for adolescents were reported as a common barrier. This was reaffirmed by both adolescents and parents, with both finding it difficult to identify phrases to search for overeating treatment availability on the internet. Similar barriers to help-seeking were reported by parents, including a lack of recognition of overeating behaviours as a problem, as well as fear of parents exacerbating the issue. While weight and eating-related stigma were reported by parents, this was not as strongly articulated as barriers compared to adolescent interviews.

Facilitators to help-seeking reported by adolescents in Stage 2 included knowledge of available services or resources, and acknowledgment of overeating as problematic. Family, friends, and health professionals were all reported to be points of access for help-seeking for adolescents affected by overeating. Generally, health professionals were perceived as more knowledgeable and qualified compared to other supports. Schools were also viewed as an important support, while it was acknowledged that friends may not always have accurate information. Interestingly, social media and influencers were reported by some adolescents to be facilitators to engaging in healthy eating behaviours and could reduce stigma or help identify overeating behaviours, assist with help-seeking, and identify when to seek help. Facilitators to help-seeking reported by parents strongly focused on the need for education, particularly in schools, as well as the availability of services. Parents agreed that a range of people and services were needed to support a young person, including school supports. Some concerns were raised about seeking help from friends or social media regarding lack of qualifications and accuracy of information, with one parent describing, *“Ongoing influence of social media making it difficult for young people to know what normal is.”*—P2.

#### 3.2.2. Subtheme 2: Overeating and Stigma

As stigma was raised as an issue in the Stage 1 interviews, we were interested in exploring this in more depth in the Stage 2 interviews and comparing stigma around overeating and restrictive eating behaviours. Overeating appeared to be perceived as more internally and externally stigmatising than restricting in many adolescents. Adolescents associated overeating with negative body image, expectations of body size and shape, shame and embarrassment, weight gain, and being judged. For example, *“I feel like people can think you’re doing something wrong if you’re overeating and you’re gaining weight, I feel like it’s frowned upon more.”*—A6. Compared to restrictive eating, overeating was perceived by adolescents as a difficult topic to broach with peers due to the stigma around overeating and weight status and the influence of socio-cultural norms, although one adolescent did not perceive this stigma, as overeating is experienced by everyone. There was a general perception that seeking help for dietary restriction would be easier, although one adolescent participant viewed these as equally problematic.

All parents believed there was a strong connection between overeating and weight stigma, specifically relating to weight and body image, as well as the influence of socio-cultural norms. Compared to overeating, parents perceived that people experiencing dietary restriction would be more likely to seek help and easier to raise as a concern in the Stage 2 interviews, for example, *“More commonly talked about to go on a diet or go on meal replacements but acknowledging that you constantly overeat would be tougher.”*—P5 and *“Saying you’re eating a lot may be more offensive… If you’re talking about overeating, it might sound more judgey or patronising as opposed to caring.”*—P7.

### 3.3. Theme 3: Perceptions of Other Eating Behaviours

#### 3.3.1. Subtheme 1: Diets, Dieting, and Restricting

To compare attitudes and understanding between overeating and other eating behaviours, we asked about dieting and restricting. When adolescents were asked about their interpretation of the word ‘diet’ or ‘dieting’ in both Stage 1 and Stage 2 interviews, there were mixed perceptions of whether this was perceived as positive or negative. Most adolescents viewed ‘diet’ or ‘dieting’ as their daily intake or what they commonly eat, *“a range of things that you typically eat”*—A1. However, some adolescents associated ‘diet’ or ‘dieting’ with restrictive or controlled eating practices; *“I hear the word diet, I think of a strict eating schedule that you follow.”*—A4, and *“It does imply some restrictiveness”*—A6. Adolescents also perceived generational differences in understanding of the term compared to their parents: *“Everyone has a diet, so that’s why I would say it is what you eat, but I feel like when you’re younger you may hear your parents use ‘diet’ in a restrictive way… lots of mums, less so dads”*—A8. In contrast, parents were more aware of ‘diet’ and ‘dieting’ as words related to weight control, specific health needs, or sporting aspirations. In the Stage 2 interviews, most parents perceived that adolescents would view ‘diet’ or ‘dieting’ as negative, associating them with restricting and weight loss. However, it was acknowledged that due to the increasing number of diets for medical reasons now, adolescents may *“have a different concept of diet because there are different types of diets, not just weight loss, whereas a generation ago they would have thought more in terms of calories and weight status.”*—P2.

Differing views were expressed regarding ‘restricting’, with some adolescent participants perceiving this as a negative behaviour associated with intentional weight loss, while others held views that restriction as a positive or negative concept depends on the types of foods that are restricted. For example, *“being restricted because they’re eating bad foods all day. But not like restricting how many carbs you eat.”*—A5. While one parent perceived that adolescents would see ‘restricting’ as *“aspirational; if they are restricting or imposing diet rules, it is something they want to follow or enforce.”*—P2, most parents believed ‘restricting’ would be perceived as negative by adolescents.

#### 3.3.2. Subtheme 2: Healthy Eating and Normal Eating

In the Stage 1 interviews, adolescents’ perceptions of ‘healthy eating’ reflected a balanced diet including a range of healthy foods. For example, *“I think healthy eating is eating a broad range of good, nutritious foods. A lot of people might think of healthy eating as cutting [out] and eating less.”*—A9, while ‘being healthy’ was reflected by adolescents as not only healthy eating, but a combination of healthy eating behaviours. Interestingly, adolescent views of ‘normal eating’ differed to that of healthy eating, reflecting more of what is normal for an individual, and may not necessarily be ‘healthy’. For example, *“Everyone has their own normal eating habits and normal eating styles… normal eating could be healthy or unhealthy.”*—A4. These differences in perceptions were supported in Stage 2 interviews. Despite adolescents making clear delineations between terms, most parents reported that they expected adolescents to perceive these terms as essentially the same, although parents were less likely to see these as synonymous.

## 4. Discussion

This study aimed to explore adolescent and parent perceptions of eating behaviours, with a focus on overeating behaviours, and associated health treatment-seeking behaviours. The major findings indicate that participants’ interpretation of overeating varied; some associated overeating with features of binge eating, while others did not perceive it as problematic, depending on the social context. However, both adolescents and parents linked problematic overeating to increased frequency of episodes and perceived that adolescents were less likely to seek help for overeating, often related to weight-related stigma, compared to restrictive eating. In addition, there were some differences in adolescent and parent understandings of eating behaviours, such as food addiction and healthy eating. These findings highlight the need for interventions that build awareness and avenues of support for overeating behaviours. They also highlight the importance of promoting a shared understanding across generations of healthy eating attitudes and behaviours. This could assist with preventing the progression from occasional to habitual overeating and reducing the risk of developing an eating disorder during adolescence.

The frequency and context of overeating were commonly linked by both parents and adolescents to the perception of whether overeating was viewed as a problematic eating behaviour, with eating during social events viewed as part of normal eating. When overeating started to impact function or become more frequent, it was perceived as more problematic; however, the frequency at which overeating behaviours transitioned to become problematic was not explicitly defined by participants. Overeating was also commonly reported in this study in connection with emotional eating or feeling down, which aligns with other research exploring overeating and mental ill health (Mills et al., 2020). Recognising when overeating behaviours are becoming problematic is particularly important to guide the development of effective prevention and early intervention interventions, which are underrepresented compared to restrictive disorders. Eating disorder mental health literacy is increasing in the general community; however, most research to date has focused on anorexia nervosa and bulimia nervosa (Bullivant et al., 2020). The findings of this study provide some insight into mental health literacy for overeating disorders; however, further research is needed to better understand perceptions of when overeating behaviours become problematic and warrant help-seeking.

Adolescents perceived overeating as a difficult topic to broach due to stigma, especially when compared to restrictive eating. Moreover, help-seeking for overeating was perceived as challenging, as adolescents lacked an awareness of available or appropriate services. Previous research supports perceived challenges of help seeking, with only approximately 10% of Australian adolescents seeking help for an eating disorder, and those with binge eating disorder were less likely to seek help compared to those with anorexia nervosa or bulimia nervosa (Fatt et al., 2019). Adolescents also found overeating more difficult to identify amongst peers than restrictive eating. Parents acknowledged stigma associated with overeating and expressed a reluctance to raise the topic of problematic eating behaviours, including overeating and restrictive eating, due to weight-related stigma. Adolescents’ internalised weight-related stigma may present a barrier to help-seeking for overeating due to the perception that they should be able to ‘control’ their eating behaviours (Romano et al., 2018). This has implications for early intervention strategies, as people may not recognise overeating as problematic and seek help until behaviours are entrenched and more difficult to change. This is in line with the COBWEBS model, which characterises weight-based stigma as a vicious cycle, with increased cortisol resulting from weight stigma leading to further weight gain and difficulty losing weight (Lee et al., 2024; Tomiyama, 2014). In addition, qualitative work has shown that many adults can identify the commencement of overeating behaviours during childhood and adolescence (Collins et al., 2020), reaffirming that adolescence is a key period for engagement and early intervention. This is particularly relevant as overeating behaviours such as binge eating have been linked to chronic physical and mental health conditions, such as diabetes, depression, and anxiety in adults (Sheehan & Herman, 2015), warranting further exploration regarding appropriate support avenues and treatment models for adolescent weight management or mental health services.

Schools were identified by parents and adolescents as places where awareness and help-seeking could be promoted, with social media also suggested by adolescents as a suitable platform for education. Previous research suggests school professionals are willing to engage in eating disorder prevention; however, inconsistencies in the perceptions of school professionals’ roles in eating disorder prevention were reported (Pursey et al., 2022). In addition, this previous work focused predominantly on restrictive eating disorders, highlighting the need to understand school professionals’ attitudes to a wider range of eating disorder behaviours, including overeating, particularly in the context of large-scale healthy lifestyle interventions in the school setting (Pursey et al., 2021). Identification and structure of school supports should be tailored within individual school settings, for example, school counsellors, wellbeing teams, and school nurses. In addition, school professionals should explore the meaning and potential differences in terminology around overeating being used by adolescents when providing support.

When exploring other terms associated with overeating, food addiction was widely recognised by adolescents and was consistently linked to problematic behaviours and loss of control. Several terms have been used to refer to food addiction, with recent research suggesting that “ultra-processed food addiction” best represents the pharmacokinetic properties of potentially addictive foods (McCausland et al., 2025). In the current study, compared to other eating behaviours, food addiction was more frequently linked to UPF foods or food components such as refined sugars, compared to binge or overeating and problematic eating. Food addiction is often operationalised using the DSM criteria for substance use disorder (i.e., substances or ingredients in food eliciting an addictive-like response) (Bonder & Davis, 2022), and this appeared to be echoed in the adolescents’ understanding of the term when associating with specific foods. However, parents did not associate the same level of severity when speaking about food addiction, reflecting a potential generational mismatch in the understanding of these terms.

Similarly, adolescents’ understanding of the term ‘binge eating’ was underestimated by parents, with most parents reporting that they did not expect adolescents to be familiar with this term. While some referenced features of DSM-5 diagnosable binge eating, such as feeling bad and overeating over a short period of time (American Psychiatric Association, 2013), others were less familiar with the term ‘binge eating’ but applied their knowledge of the word binge in other contexts (e.g., TV) to eating. Given that several tools, including the EDE-Q, refer to the term binge (Fairburn et al., 2008) in the context of food, suggests that there needs to be more of a shared understanding of the term ‘binge eating’, particularly in the context of written screening and assessment questionnaires. The findings of this study reinforce the importance of patient-centred care, including consideration of stigma-sensitive language. Future qualitative research should explore stigma-sensitive recommendations in more detail, for example, language to avoid, ways to separate moral judgment from behaviour description, and how to engage parents without intensifying shame. It is recommended that health professionals check the meaning and understanding of terms used by adolescents and their parents to describe eating behaviours.

Differences in the interpretations of ‘healthy’ compared to ‘normal’ eating demonstrated these terms were not viewed as synonymous by adolescents, which contrasted with parent perceptions. Of note, healthy eating was also associated with “strict” eating and “good foods”, which may reflect an interpretation of highly regimented eating. Similar discrepancies have been reported between parents and adolescents in their perceptions of diet and physical activity in the context of weight management interventions in the US (Azar et al., 2020). However, the current study recruited adolescents and mothers from the general population, in contrast to the weight management setting in the US study. In our study, parents perceived the term ‘diet’ to reflect a specific eating plan which may include restrictive practices, while adolescents tended to report ‘dieting’ as a neutral term, simply reflecting what is eaten throughout the day. Often in clinical practice, public health initiatives, and education curricula in schools aimed at adolescents, the terms ‘healthy eating’ and ‘diet’ are used. Having awareness of these generational differences highlights the need to consider interpretation when striving to intervene in adolescent health behaviours, for example, considering parental vs. adolescent reports in screening and assessment tools.

Limitations of this study include the over-representation of female adolescents; therfore, the results may not be generalisable to other groups. Furthermore, all parents were female and identified as mothers; therefore, the results may be different from those of other caregivers, including paternal caregivers. This gender distribution likely influences how eating is moralised, who initiates help-seeking, and which vocabularies feel socially acceptable. There was a low retention rate in the Stage 2 interviews, which may have affected data saturation during this stage. However, the Stage 2 interviews reinforced existing themes and data quality, rather than introducing new perspectives, suggesting data sufficiency was reached. Recruitment via professional networks may have contributed to sampling bias in recruiting individuals already connected to care or those more comfortable in discussing eating behaviours. No adolescents reported having experienced problematic eating behaviours themselves in the demographic survey; therefore, views may differ from those with lived experience of problematic eating behaviours. The positionality of the interviewers and coders as dietitians may have shaped participant responses, for example, moral framing and social desirability. There was also potential for researcher confirmation bias, although this was mitigated by researchers independently coding the data before meeting to discuss themes, along with additional discussion with the broader research team. The interviews were conducted during a period affected by COVID-19. Isolation, increased screentime, and altered exposure to food and food-related messaging related to COVID-19 restrictions may have influenced responses in this study. Demographic information, including socioeconomic status, ethnicity, and geographical location, as well as anthropometric measurements, were not collected as part of this study due to the qualitative, exploratory nature of the study. Future studies should consider collecting this information, as these factors may influence eating behaviours and views of such behaviours.

## 5. Conclusions

This study identified that there are differences between adolescents and their parents in perceptions of overeating and associated terminology, as well as other eating behaviours. This reflects a need for a shared understanding of terms associated with overeating between clinicians, adolescents, and parents. This should also be considered with tools to assess eating behaviours, including overeating, healthy eating, and dieting, to ensure adolescent comprehension, and that their responses accurately reflect eating behaviours. Due to these potential differences in understanding of terminology, in clinical settings, health professionals should explore adolescents’ understanding of terms to describe overeating behaviours and individualise communication appropriately to match this. The qualitative study identified that stigma is a significant issue in help-seeking for overeating in adolescence, highlighting the need for clearer clinical service entry points and conversation starters for adolescents experiencing overeating. Future research should explore perceptions of overeating evolving from normal social-related behaviours to becoming problematic to inform the development of timely prevention and early intervention interventions.

## Data Availability

Data available upon reasonable request from the authors.

## Supplementary Materials

The following supporting information can be downloaded at: https://www.mdpi.com/article/10.3390/bs16030328/s1, Table S1: Stage 1 Interview Schedule; Supplementary data: Stage 1 themes and identified nine elements to be addressed during the Stage 2 interviews.
