# Supplementary material for: Decoding Adolescents’ and Parents’ Perspectives of Overeating: A Qualitative Study
Source: Behav Sci (Basel). 2026 Feb 27;16(3):328. doi: 10.3390/bs16030328 (PMC13023448; doi:10.3390/bs16030328)
Supplement: Supplementary file 1 [file behavsci-16-00328-s001.zip › behavsci-4077354-supplementary.pdf]

**Supplementary Materials**

Table S1. Stage 1 Interview Schedule

| | Adolescents | Parents/caregivers |
|---|---|---|
| **Terminology** | Thinking of terms associated with eating and the different ways of eating, what words or phrases would you or your peers use to look for information about overeating? | Thinking of terms associated with eating and the different ways of eating, what words or phrases do you think your child, or their peers use to look for information about overeating? |
| | • What is your impression or understanding of what the word 'overeating' means?<br>• What is your impression or understanding of what the word 'diet' or 'dieting' means?<br>• What is your impression or understanding of what the word 'food addiction' means?<br>• What is your impression or understanding of what 'out of control eating' means?<br>• What is your impression or understanding of what the word 'emotional eating' means?<br>• What is your impression or understanding of what the word 'fasting' means?<br>• What is your impression or understanding of what the word 'healthy eating' means?<br>• What is your impression or understanding of what the word 'normal eating' means? | • What do you think your child's impression or understanding of what the word 'overeating' means?<br>• What do you think your child's impression or understanding of what the word 'diet' or 'dieting' means?<br>• What do you think your child's impression or understanding of what the word 'food addiction' means?<br>• What do you think your child's impression or understanding of what 'out of control eating' means?<br>• What do you think your child's impression or understanding of what the word 'emotional eating' means?<br>• What do you think your child's impression or understanding of what the word 'fasting' means?<br>• What do you think your child's impression or understanding of what the word 'healthy eating' means?<br>• What do you think your child's impression or understanding of what the word 'normal eating' means? |
| **Views/perceptions about terminology** | • overeating<br>• dieting<br>• healthy eating or 'being healthy' | • overeating<br>• dieting<br>• healthy eating or 'being healthy' |

| | | |
|---|---|---|
| | • bingeing<br>• dietary restricting<br>• fasting<br>• dietary rules<br>• night eating<br>• large amounts of food<br>• what is normal eating<br>• ‘out of control’ eating<br>• emotional eating<br>• guilt around eating<br>• food addiction<br>• addicted to food | • bingeing<br>• dietary restricting<br>• fasting<br>• dietary rules<br>• night eating<br>• large amounts of food<br>• what is normal eating<br>• ‘out of control’ eating<br>• emotional eating<br>• guilt around eating<br>• food addiction<br>• addicted to food |
| **Beliefs** | Thinking of the words we just talked about, which ones of these behaviours or feelings would seem ok and ‘normal’ if you or a friend were experiencing them? | Thinking of the words we just talked about, which ones of these behaviours or feelings do you think your child would seem ok and ‘normal’ if they or a friend were experiencing them? |
| | Thinking about the words we just talked about, are there times when some of these terms/behaviours are normal and ok? | Thinking about the words we just talked about, are there times when your child would think some of these terms/behaviours are normal and ok? |
| | When do you think it would be enough of an issue that <u>you</u> would, or you would tell a friend to seek help? | When do you think it would be enough of an issue that <u>your child</u> would seek help, or would tell a friend to seek help? |
| **Treatment/ health seeking** | Where do you think you would get help from or suggest to a friend to seek help? | Where do you think they would get help from or suggest to a friend to seek help? |
| | Can you think of anything that would get in the way of seeking help? | Can you think of anything that would get in the way of them seeking help? |

| | | |
|---|---|---|
| | Can you think of anything that would get in the way of receiving help? | Can you think of anything that would get in the way of them receiving help? |
| | Can you think of things that would make it easier to get help? | Can you think of things that would make it easier for them to get help? |

**Supplementa1 data:** Stage 1 themes and identified nine elements to be addressed during the Stage 2 interviews

(1) similarities and differences between ‘healthy eating’ and ‘normal eating’

(2) acceptability of the terminology associated with food addiction

(3) interpretations of the words ‘diet’ and ‘dieting’

(4) whether ‘restricting’ was viewed as negative or positive

(5) associations between overeating and boredom

(6) overeating and restricting stigma

(7) help seeking for overeating

(8) roles of family, friends, schools and health professionals in supporting a young person affected by overeating

(9) any perceived differences between how adolescents and their parents view food and eating.
